# Distal Cholangiocarcinoma with Synchronous Intramural Bile Duct Metastasis: A Case Report

**DOI:** 10.70352/scrj.cr.25-0119

**Published:** 2025-07-11

**Authors:** Ryota Kiuchi, Hitaru Ishioka, Tomohiro Akutsu, Mitsumasa Makino, Hisato Ishimatsu, Masanori Yamazaki, Tsuyoshi Shoji, Rui Nomura, Yutaka Kurebayashi, Hirotoshi Maruo

**Affiliations:** 1 Department of Surgery, Shizuoka City Shimizu Hospital, Shizuoka, Shizuoka, Japan; 2 Department of Diagnostic Pathology, Shizuoka City Shimizu Hospital, Shizuoka, Shizuoka, Japan; 3 Department of Pathology, Keio University School of Medicine, Tokyo, Japan

**Keywords:** distal cholangiocarcinoma, intramural bile duct metastasis

## Abstract

**INTRODUCTION:**

Distal cholangiocarcinoma is a malignant tumor that arises from the epithelial cells of the bile duct. Several risk factors associated with cholangiocarcinoma have been identified. Multiple distal cholangiocarcinomas may occur in patients with several risk factors for cholangiocarcinoma. However, synchronous multiple distal cholangiocarcinomas in the absence of risk factors are rare. Here, we presented a case of multiple tumors on the bile duct diagnosed as distal cholangiocarcinoma with synchronous intramural bile duct metastasis.

**CASE PRESENTATION:**

A 67-year-old man was referred to our hospital for evaluation of jaundice. Contrast-enhanced computed tomography revealed an enhanced tumor on the common bile duct. Endoscopic retrograde cholangiography showed bile duct stenosis due to a nodular tumor of the common bile duct. We performed subtotal stomach-preserving pancreaticoduodenectomy under diagnosing distal cholangiocarcinoma. The patient was discharged on the 23rd postoperative day. Macroscopic findings of the resected specimen showed a 27-mm nodular-infiltrating tumor at the middle bile duct and a 3-mm nodular tumor at the lower bile duct. The distance between the tumors was 10 mm. Pathological examination revealed that the larger tumor was primarily composed of poorly differentiated adenocarcinoma, with a moderately differentiated component at the periphery of the tumor. The smaller tumor was entirely composed of poorly differentiated adenocarcinoma, which was similar to the poorly differentiated component of the larger tumor. Additionally, microscopic lymphovascular infiltration was observed in the vicinity of both tumors. These two lesions were separated by nontumoral biliary epithelia without atypia. The results of immunohistochemical staining using CK7/20, MUC1/2, and p53 antibodies substantiated the homology of these tumors. These results suggested that the smaller tumor was synchronous intramural bile duct metastasis of distal cholangiocarcinoma rather than independent multiple lesions.

**CONCLUSIONS:**

In the cases of multiple tumors being synchronously identified on the bile duct, it is crucial to ascertain the relationship between those tumors. Recent developments in immunohistochemical staining and genetic analysis may further facilitate the assessment of the association between multiple distal cholangiocarcinomas.

## Abbreviation

HE
: hematoxylin and eosin

## INTRODUCTION

Extrahepatic cholangiocarcinoma is a malignant tumor that arises from the epithelial cells of the extrahepatic bile duct. Extrahepatic cholangiocarcinoma is classified into perihilar and distal cholangiocarcinoma according to the anatomical location of the tumor. Several risk factors associated with distal cholangiocarcinoma have been identified, including pancreaticobiliary maljunction^1,2)^ and exposure of 1,2-dichloropropane.^3,4)^ Although multiple distal cholangiocarcinomas may occur in patients with these risk factors, most distal cholangiocarcinomas occur as a solitary lesion. The occurrence of synchronous multiple distal cholangiocarcinomas in patients without these risk factors is rare. Additionally, it is crucial to identify the relationship between these multiple distal cholangiocarcinomas.

Herein, we presented a case of distal cholangiocarcinoma with synchronous intramural bile duct metastasis occurring in the absence of any identifiable risk factors.

## CASE PRESENTATION

**Fig. 1:**
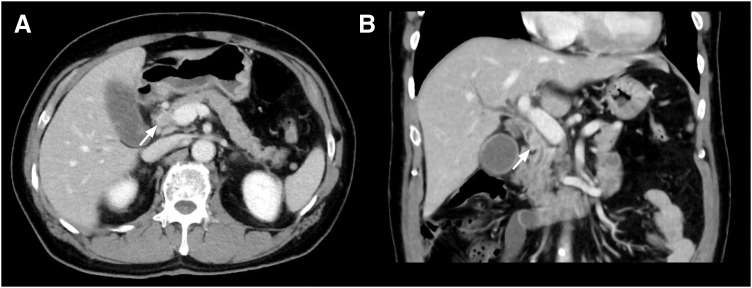
Findings of contrast-enhanced CT. Contrast-enhanced CT revealed a well-enhanced tumor in the middle bile duct (arrow) in the axial image (**A**) and coronal image (**B**).

**Fig. 2:**
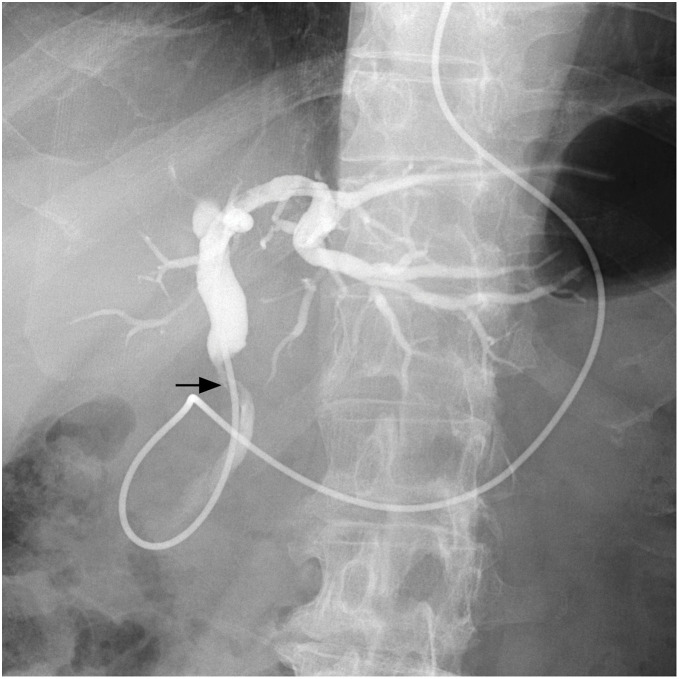
Findings of cholangiography. Cholangiography using a nasal biliary tube revealed a contrast defect in the middle bile duct (arrow). No evidence of pancreaticobiliary maljunction was found.

A 67-year-old man presented to a local physician with a fever. Physical examination revealed scleral icterus, and the laboratory test showed hyperbilirubinemia and an elevated C-reactive protein level. He was referred to our hospital for a comprehensive examination and subsequent treatment. Contrast-enhanced computed tomography revealed an enhanced tumor on the common bile duct and intrahepatic bile duct dilation (**Fig. 1**). Cholangiography using a nasal biliary tube showed the bile duct stenosis due to the nodular tumor of the common bile duct (**Fig. 2**). Cholangiography did not confirm the evidence of pancreaticobiliary maljunction. Biopsy of the bile duct tumor diagnosed moderately differentiated adenocarcinoma. Preoperative radiological examinations detected only one lesion on the distal bile duct. After jaundice improved with retrograde biliary drainage, subtotal stomach-preserving pancreaticoduodenectomy was performed under diagnosing distal cholangiocarcinoma. Although postoperative pancreatic fistula in biochemical leaks, classified by the International Surgical Group of Pancreatic Fistula,^5)^ developed, the postoperative pancreatic fistula was managed conservatively. The patient was discharged on the 23rd postoperative day.

**Fig. 3:**
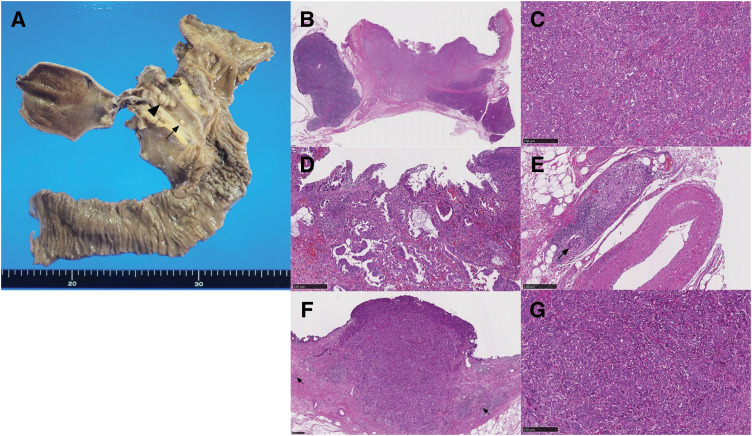
Findings of pathological examinations. (**A**) Macroscopic findings of resected specimen. Two lesions were identified on the bile duct. The larger lesion was situated at the middle bile duct and measured 27 mm in diameter (arrowhead). The smaller lesion was located at the lower bile duct and measured 3 mm in diameter (arrow). The distance between those tumors was 10 mm. Macroscopic findings showed no continuity between those lesions. (**B**) Findings of loupe image of larger distal cholangiocarcinoma (HE staining). (**C**) The poorly differentiated adenocarcinoma component was located at the center of the larger distal cholangiocarcinoma (HE staining, ×100). (**D**) The moderately differentiated adenocarcinoma component was located at the margin of the larger distal cholangiocarcinoma (HE staining, ×100). (**E**) Microscopic lymphovascular infiltration of the larger distal cholangiocarcinoma (arrow) (HE staining, ×100). (**F**) Findings of loupe image of smaller distal cholangiocarcinoma (HE staining, ×40). The smaller distal cholangiocarcinoma was located in the mucosal lamina propria. Additionally, microscopic lymphovascular infiltration was observed in the vicinity of the smaller tumor (arrow). (**G**) The smaller distal cholangiocarcinoma was entirely composed of poorly differentiated adenocarcinoma, analogous to that of the larger distal cholangiocarcinoma (HE staining, ×100). HE, hematoxylin and eosin

**Fig. 4:**
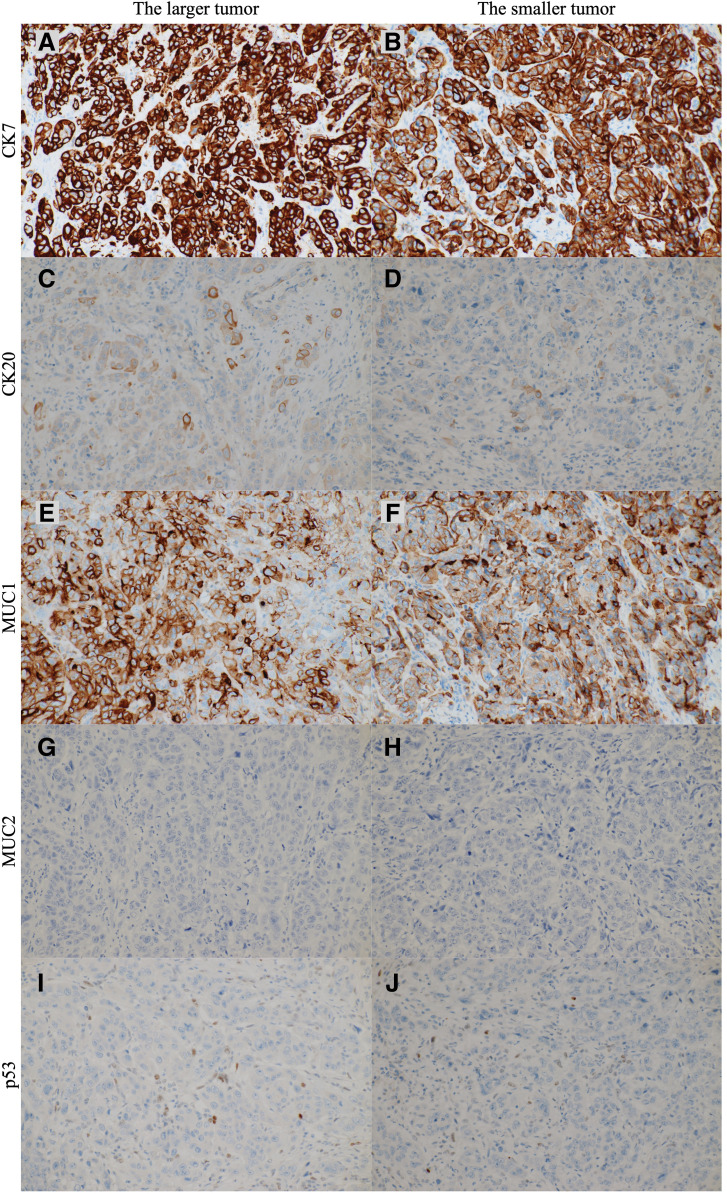
Findings of immunohistochemical staining examinations in larger and smaller distal cholangiocarcinomas. (**A**, **B**) Findings of immunohistochemical staining for CK7 (×200). The tumor cells in both tumors were strongly positive for CK7. (**C**, **D**) Findings of immunohistochemical staining for CK20 (×200). The tumor cells in both tumors were focally positive for CK20. (**E**, **F**) Findings of immunohistochemical staining for MUC1 (×200). The tumor cells in both tumors were strongly positive for MUC1. (**G**, **H**) Findings of immunohistochemical staining for MUC2 (×200). The tumor cells in both tumors were negative for MUC2. (**I**, **J**) Findings of immunohistochemical staining for p53 (×200). The staining patterns for p53 were wild pattern in both tumors. These results of immunohistochemical staining revealed the homology between larger and smaller distal cholangiocarcinomas.

Macroscopic findings of the resected specimen showed a 27-mm nodular-infiltrating tumor at the middle bile duct and a 3-mm nodular tumor at the lower bile duct (**Fig. 3A**). The distance between those tumors was 10 mm. Pathological examinations revealed that the larger tumor was primarily composed of poorly differentiated adenocarcinoma, with a moderately differentiated component at the periphery of the tumor (**Fig. 3B**–**3D**). The invasive tumor thickness was 8 mm. Microscopic lymphovascular infiltration was observed (**Fig. 3E**). The smaller tumor was located within the submucosal layer (**Fig. 3F**). The smaller tumor was entirely composed of poorly differentiated adenocarcinoma, which was similar to the poorly differentiated component of the larger tumor (**Fig. 3G**). Additionally, microscopic lymphovascular infiltration was observed in the vicinity of the smaller tumor (**Fig. 3F**). These two tumors were completely separated by non-tumoral biliary epithelia, without atypia. These pathological results suggested that the smaller tumor was diagnosed as an intramural bile duct metastasis of distal cholangiocarcinoma via microscopic lymphovascular infiltration, rather than multiple independent lesions. The immunohistochemical staining was performed using CK7/20, MUC1/2, and p53 antibodies (**Fig. 4**). The tumor cells in both the larger and smaller lesions exhibited strongly positive for CK7 and MUC1, focally positive for CK20, and negative for MUC2. The staining pattern for p53 was wild pattern in both tumor cells. These results of immunohistochemical staining substantiated the homology between both lesions. No lymph node metastasis was observed. Using the UICC TNM classification, 8th edition, the tumor was diagnosed as T2N0M0 Stage IIA.^6)^ Although adjuvant chemotherapy with S1 was completed, multiple liver metastases occurred in the 7th month after surgery.

## DISCUSSION

Extrahepatic cholangiocarcinoma is a malignant tumor that arises from the epithelial cells of the extrahepatic bile duct. Extrahepatic cholangiocarcinoma is classified into perihilar or distal cholangiocarcinoma according to the anatomical location of the tumor. The incidence of distal cholangiocarcinoma varies from 0.53 to 2 cases per 100,000 person-years worldwide.^7)^ Several risk factors have been identified to be associated with distal cholangiocarcinoma, including pancreaticobiliary maljunction^1,2)^ and exposure of 1,2-dichloropropane.^3,4)^ Multiple distal cholangiocarcinomas may develop in patients with these risk factors. There have been several reports of metachronous cholangiocarcinoma after curative resection.^8–10)^ Including our case, seven cases of synchronous multiple cholangiocarcinomas in the absence of these risk factors have been reported^11–16)^ (**Table 1**). Among these cases, three cases were diagnosed as double primary cancers, two cases did not describe the relationship between the tumors in detail, and only two cases, including our case, were diagnosed as synchronous intramural bile duct metastasis. These results would underscore the rarity of distal cholangiocarcinoma with synchronous intramural bile duct metastasis.

**Table 1:** Reported cases of synchronous multiple extrahepatic cholangiocarcinoma without pancreaticobiliary malfunction

Author	Age	Gender	Surgical procedure	Larger tumor location	Larger tumor pathological results	Smaller tumor location	Smaller tumor pathological results	Immunohistological staining	Genetic analysis	Primary or metastasis	Prognosis
Ogawa et al.^14)^	69	Male	PPPD	Middle bile duct	Por	Inferior bile duct	Mod	None	PCR	Synchronous metastasis	Not described
Bedoui et al.^11)^	67	Female	PPPD	Middle bile duct	Not described	Inferior bile duct	Not described	None	None	Not described	Not described
Sumiyoshi et al.^15)^	78	Male	1st EHBD, 2nd PD	Perihilar bile duct	Mod	Inferior bile duct	Pap	None	None	Not described	31 months, alive
Yoo et al.^16)^	67	Male	PPPD	Middle bile duct	Mod	Inferior bile duct	SCC	None	None	Double primary	8 months, dead
Nishi et al.^13)^	78	Female	PPD	Middle bile duct	Wel	Inferior bile duct	Por	CD56, chromogranine A, synaptophysin	None	Double primary	18 months, dead
Morita et al.^12)^	69	Male	RHPD	Perihilar bile duct	Mod	Inferior bile duct	Por	p53	PCR-SSCP	Double primary	28 months, alive
Our case	67	Male	SSPPD	Middle bile duct	Por and mod	Inferior bile duct	Por	CK7, CK20, MUC1, MUC2, p53	None	Synchronous metastasis	7 months, alive

EHBD, extrahepatic bile duct resection; Mod, moderately differentiated adenocarcinoma; Pap, papillary adenocarcinoma; PCR, polymerase chain reaction; PCR-SSCP, polymerase chain reaction-single-stand conformation polymorphism; PD, pancreaticoduodenectomy; Por, poorly differentiated adenocarcinoma; PPPD, pylorus-preserving pancreaticoduodenectomy; RHPD, right hepatopancreatidoduodenectomy; SCC, squamous cell carcinoma; SSPPD, subtotal stomach preserving pancreaticoduodenectomy; Wel, well differentiated

In the case of synchronous multiple tumors being identified in the same organ, it is important to determine whether these tumors are diagnosed as multiple primary tumors. Multiple primary cancers have been defined according to the following criteria: (1) each tumor must present a definite picture of malignancy, (2) each tumor must be distinct, and (3) the probability that one tumor is the metastasis of the other must be ruled out.^17)^ In the context of extrahepatic cholangiocarcinoma, the relationship between multiple tumors should be considered in light of four proposed mechanisms: (1) intraductal spread of neoplastic cells via carcinoma *in situ*, (2) intramural metastasis via lymphovascular or perineural infiltration, (3) intramural seeding of neoplastic cells via bile juice, and (4) multicentric carcinogenesis in the setting of field carcinogenesis.^18)^ In our case, no identifiable risk factors for the development of cholangiocarcinoma were found. No evidence of carcinoma *in situ* was observed between the two lesions. The smaller tumor was primarily located under mucosal layer, making intramural seeding via bile juice an unlikely mechanism. Microscopic lymphovascular infiltrations were observed in the vicinity of both tumors. Furthermore, the smaller tumor, composed of poorly differentiated adenocarcinoma, resembled the larger tumor. Based on these pathological findings using hematoxylin and eosin (HE) staining, the case was diagnosed as distal cholangiocarcinoma with synchronous intramural bile duct metastasis.

Recent advances in immunohistochemical and genetic analyses have helped us evaluate the relationships between the multiple cholangiocarcinomas. Immunohistochemical staining of p53 protein and polymerase chain reaction-single-strand conformation polymorphism of *TP53* gene aided in the evaluation of synchronous double cholangiocarcinomas as double primary cancers.^12)^ The loss of heterozygosity assay revealed the synchronous double cholangiocarcinomas as intramural bile duct metastasis.^14)^ Next-generation sequencing has been recently used to analyze the genomics of extrahepatic cholangiocarcinomas.^19,20)^ A comprehensive analysis of the molecular characteristics of primary and metachronous cholangiocarcinomas using next-generation sequencing has elucidated the pathways and mechanisms underlying multiple cholangiocarcinomas.^18)^ Comprehensive analysis revealed the three distinct molecular pathways: (1) the successional pathway, (2) the phylogenic pathway, and (3) the distinct pathway. Although genetic analyses were not performed in this case, immunohistochemical staining examination was conducted. The results of immunohistochemical staining using CK7/20, MUC1/2, and p53 antibodies revealed the homology between the larger and smaller tumors, and would substantiate the diagnosis established by the results from HE staining. These validation analyses including next-generation sequencing would have been possible if the advanced inspections could be made readily available.

The optimal treatment for extrahepatic cholangiocarcinoma is surgical resection, such as pancreaticoduodenectomy or hepatectomy with bile duct resection.^21,22)^ Several articles have reported aggressive reoperations for metachronous cholangiocarcinoma at the remnant bile duct following curative resection. Although reoperations after curative resection for cholangiocarcinoma require highly proficient surgical skills to secure sufficient surgical margins, these procedures with negative surgical margins can result in a favorable prognosis.^8,18,23)^ The benefits of surgical resection for distal cholangiocarcinoma with synchronous intramural bile duct metastasis remain unclear because of the rarity of this condition and the possibility of high malignancy due to the presence of metastasis at the time of resection. In this case, multiple liver metastases occurred in the 7th month after surgery. Nevertheless, curative surgical resection could contribute to prolong survival in patients accompanied by distal cholangiocarcinoma with synchronous intramural bile duct metastasis.

## CONCLUSIONS

We experienced a patient of distal cholangiocarcinoma with synchronous intramural bile duct metastasis diagnosed based on the findings from HE and immunohistochemical staining. It is crucial to ascertain the relationship between these tumors in the cases of multiple distal cholangiocarcinomas being identified. Recent developments in immunohistochemical techniques, genetic analyses, and next-generation sequencing can facilitate assessment of the association between multiple distal cholangiocarcinomas.

## DECLARATIONS

### Funding

This study did not receive any funding.

### Authors’ contributions

RK was involved in the clinical practice, conception, design, and acquisition of data.

HI, MM, RN, YK, and HM were involved in the clinical practice and conception and design.

TA, HI, MY, and TS approved the final version of the manuscript.

All authors have read and approved the manuscript, and they are responsible for the manuscript.

### Availability of data and materials

The datasets used in this study are available from the corresponding author upon reasonable request.

### Ethics approval and consent to participate

This work does not require ethical considerations or approval. Informed consent to participate in this study was obtained from the patient.

### Consent for publication

Informed consent was obtained from the patient for publication of this case report.

### Competing interests

All authors declare that they have no conflict of interest.
